# Enhancing flood evacuation behaviour through Serious Disaster Game (SDG): Flood Alert!

**DOI:** 10.4102/jamba.v18i1.2005

**Published:** 2026-06-09

**Authors:** Furqan I. Aksa, Muhammad Ashar, Heni W. Siswanto

**Affiliations:** 1 Department of Geography Education, Faculty of Education, Universitas Samudra, Langsa, Indonesia; 2 Department of Informatic Engineering, Faculty of Engineering, Universitas Negeri Malang, Malang, Indonesia; 3 Center for Educational Research, Faculty of Social Sciences and Humanities Research Organization, National Research and Innovation Agency (BRIN) Republic of Indonesia, Jakarta, Indonesia

**Keywords:** disaster game, flood, evacuation, hazard, disaster

## Abstract

Disaster education has been using a conventional module-based approach, which is only a one-way information transfer process. This research aims to develop and test the Serious Disaster Games (SDG): Flood Alert to improve flood evacuation behaviour and intrinsic motivation as measured by two indicators, namely knowledge about best evacuation practices and self-efficacy in dealing with flood emergencies. This research used a one-group pre-test–post-test design involving 45 students. The results showed that the SDG: Flood Alert effectively improved students’ knowledge and self-efficacy about flood disasters. Most students were able to respond well to actions before, during and after a flood event. Students are confident that they can effectively respond during flooding emergencies. Students with high self-efficacy tend to take protective actions in the face of disasters. The result of the intrinsic motivation assessment found that students enjoy learning by using the SDG: Flood Alert!

**Contribution:**

This study contributed towards enhancement of students’ knowledge regarding flood disasters. It was achieved by using the SDG: Flood Alert, which is designed using experiential learning cycle theory, and provides a fun learning experience for students. The SDG: Flood Alert has the potential to be used as a flood disaster learning media for students in flood-prone areas.

## Introduction

Indonesia has a very high flood disaster risk index (Marfai, Sekaranom & Ward 2015; Yamamoto, Sayama & Apip 2021). The data from the National Disaster Management Authority (BNPB) of the Republic of Indonesia states that from 2014 to 2023, flood disasters occupied the highest percentage, with a total of 8334 events. In the future, the probability of flood disasters is expected to increase. This is due to global climate change, which causes an increase in extreme rainfall in most parts of Indonesia (Yamamoto et al. 2021).

Therefore, efforts are needed to reduce the impact of flood disaster risk. One of them is to encourage the right behaviour before, during and after a flood disaster (Toyoda & Tanwattana 2023). This can be done with flood disaster simulations that are carried out regularly. Furthermore, disaster education has been recognised as playing an important role in increasing the knowledge and capacity to reduce the impact of disasters (Gampell et al. 2021; Novak, Lozos & Spear 2019; Oktari et al. 2015). Scientific evidence also shows that cognitive activities during education will have long-term effects on human neurological function (Eslinger et al. 2009). This will change the way educated individuals think, reason and solve problems (Baker et al. 2011). Disaster education is believed to motivate people, especially students, to take disaster preparedness actions (Hoffmann & Muttarak 2017).

However, disaster education generally follows the cognitive learning model (Tatebe & Mutch 2015; Yasuda, Muramoto & Nouchi 2018). Teachers only transfer basic conceptual and theoretical knowledge of disaster prevention. The knowledge includes how disasters occur and the impacts of disasters. Unfortunately, it does not effectively increase students’ motivation to take preparedness actions (Muzenda-Mudavanhu, Manyena & Collins 2016; Shaw, Kobayashi & Kobayashi 2004; Yildiz, et al, 2021). In addition, conceptual learning is also not relevant to the objectives of disaster education as described in the Sendai Framework for Disaster Risk Reduction conceptual framework, which emphasises knowledge, skills and attitude (Kelman & Glantz 2015).

Tsai et al. (2015b) argued that disaster education has been using a conventional module-based approach, which is based on one-way information transfer process. This learning approach often has low emotional involvement, and it does not have an impact on behavioural change (Feng et al. 2020). In addition, disaster education has also not combined knowledge with action (Aksa et al. 2025). Previous researchers have found that the traditional learning approach has been recognised as limited and ineffective (Mossoux et al. 2016). It was argued that lecture and recitation learning methods generally do not have a significant impact on student learning outcomes (Kankanamge et al. 2020).

Therefore, a new pedagogical approach is needed to make disaster learning fun for students. Game-based learning is considered a good method to enable students to use the skills and thought processes they have learned to take appropriate actions during a disaster (Galbusera et al. 2022; Tsai et al. 2019). Serious Disaster Games (SDG) contribute positively to the learning process because they are heuristic (Mitsuhara et al. 2013; Mossoux et al. 2016; Tsai et al. 2019). Players can experience complex situations that are virtually illustrated (Mossoux et al. 2016). Game-based learning has a significant influence on education (Tsai et al. 2015a). Many researchers recognise game-based learning as having great potential when compared to traditional teaching and learning methods (Gampell et al. 2021; Kankanamge et al. 2020; Tanwattana & Toyoda 2018). Researchers assert that game-based learning can increase student engagement in learning as well as increase disaster awareness (Tsai et al. 2019). However, so far, there is very limited research when it comes to developing and implementing game-based disaster learning, especially in Indonesia. This study aims to develop and test SDG: Flood Alert! in improving evacuation behaviour.

### Game design (serious disaster game: Flood Alert!)

**FIGURE 1:**
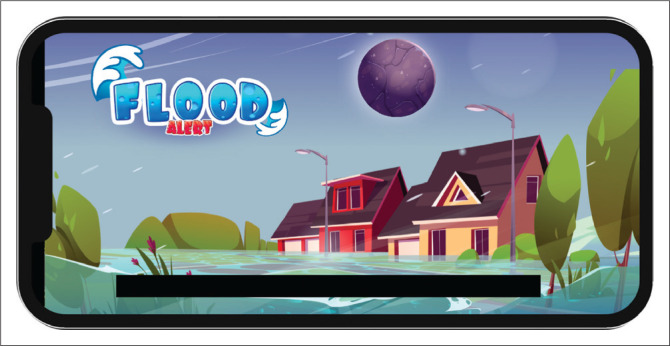
Gameplay screen.

The SDG: Flood Alert is designed to have a mission to complete a challenge in preparation when facing a flood, preparedness when a flood occurs and what to do after a flood (Figure 1). The player will enter a level, and then there is a list of commands to carry out missions such as taking items or storing important items, preparing first aid kits and finding the evacuation routes. When failing to carry out the mission, it will return to the original level. When completing all missions, the player will get points and go to the next level.

**FIGURE 2:**
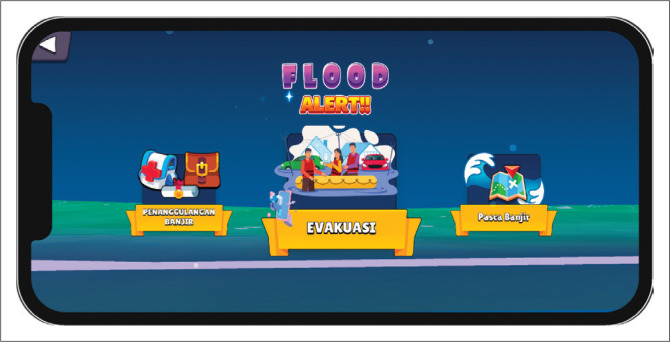
Main menu.

Serious Disaster Game: Flood Alert is designed with three levels, namely the level before the flood, the level during the flood and the level after the flood (Figure 2). In the pre-flood level, players are given the task to do several actions, namely: (1) identifying the nearest evacuation route, (2) storing important documents in a safe place, (3) preparing a disaster preparedness kit and (4) being involved in flood evacuation training.

**FIGURE 3:**
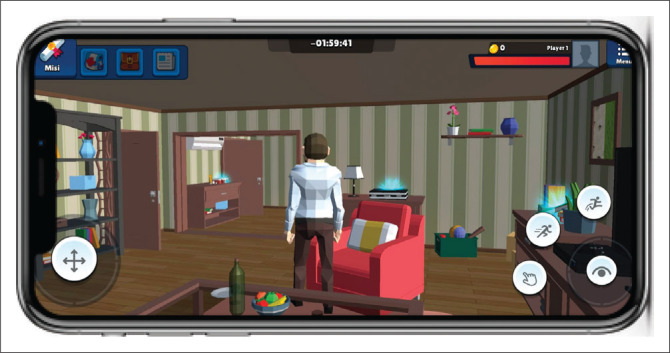
Game display.

At this level during the flood, players will be given the task of taking evacuation actions to higher grounds (when evacuating, player should not pass through the undercurrents, waterways, puddles and flooded places), preparing clean water reservoir, turning off all electricity networks during floods, not touching electrically charged equipment and not driving motorised vehicles in the flood area when the water discharge starts to rise (Figure 3). The game will fail when the player does not prepare clean water reservoirs, does not turn off the power grid, touches electrically charged equipment, walks in undercurrent or waterways and drives vehicles in flood areas.

In the level after the flood, a player will be given the task to take action to return home after an order from the authorities, dispose of food contaminated with floodwater, clean the residence and home environment from the remaining dirt after the flood, eradicate mosquito nests and be aware of electrical installations. The game will fail when the player does not complete the assigned tasks or return home before the authority’s order. The gameplay refers to the flood disaster preparedness guidebook developed by the National Disaster Management Agency (BNPB) of the Republic of Indonesia in 2019.

## Research methods and design

### Research design

This research used a one-group pre-test–post-test design. The pre-test measurement was conducted before the learning using SDGs: Flood Alert! Post-test was conducted after the application of SDGs: Flood Alert! The research involved 45 students (16 males and 29 females) from the Geography Education Study Programme at Universitas Samudra. The research was in Langsa City, which has a very high flood disaster risk index in Indonesia. The National Disaster Management Authority (BNPB) of the Republic of Indonesia points out that the number of people at risk of being affected by flood disasters reaches 125.107 individuals, with economic losses reaching 14.140 billion Rupiah.

The high risk of floods in Langsa is because the area is located at an altitude of 0 m – 25 m above sea level. Langsa City is an alluvial lowland, with rainfall intensity reaching 2300 mm per year, and high risk of flooding. These conditions cause Langsa City to experience flood disasters every year (Badan Nasional Penanggulangan Bencana Daerah Kota Langsa, 2015). Some of the worst floods occurred between 19 and 26 December 2014. This incident resulted in two deaths, evacuated 3.411 families and caused huge economic losses. In the future, the probability of flood disasters is expected to increase. The results of the hydrological study and analysis of the cross-sectional capacity of the Langsa River showed that all the river channels experienced flood conditions (overflow) (Syahputra 2015). This is caused by the flood water elevation exceeding the bank elevation (Syahputra 2015). The peak discharge at the Langsa River outlet is 59.3 m^3^/s for a 2-year return period (Syahputra 2015). These data predict that the flood disaster in Langsa City will experience a return period every 2 years (Syahputra 2015). In addition, climate change (high rainfall and sea level rise), land subsidence and sedimentation of river channels due to erosion in upstream areas also increase the probability of floods in Langsa.

### Measuring

The effectiveness of using SDGs: Flood Alert in improving evacuation behaviour response is measured by two indicators, namely knowledge about best evacuation practices, and self-efficacy in dealing with flood emergencies. The knowledge questionnaire was developed from the pocketbook Guide to Dealing with Flood Disasters, developed by the National Disaster Management Authority (BNPB) of the Republic of Indonesia in 2019.

The knowledge measurement was conducted using open-ended questions to avoid biased answers from participants. The respondents were asked the same question verbally before and after the learning session using SDG: Flood Alert! Table 1 shows assessed knowledge aspects, open-ended questions and knowledge scale.

**TABLE 1:** Assessed knowledge aspects, open-ended questions for pre- and post-training and knowledge scale.

Number	Questions	Strong knowledge	Adequate knowledge	Weak knowledge	No knowledge
1	What did you do before the flood?	4 points for knowing to: identify the nearest evacuation route, prepare a disaster preparedness kit, keep important documents in a safe place.	3 points for knowing to identify the evacuation route and prepare a disaster preparedness kit	2 points for knowing how to look for the evacuation route	1 point for knowing nothing
2	What did you do during the flood?	4 points for knowing to: evacuate to higher ground, turn off all electricity, not pass through undercurrents, drains, puddles and waterlogged places when evacuating.	3 points for knowing to: evacuate to higher ground, turn off all electricity	2 points for knowing to evacuate to higher ground	1 point for knowing nothing
3	What did you do after the flood?	4 points for knowing to: return home after an order from the authorities, dispose of food contaminated with flood water, clean the residence and home environment from residual dirt after flood, eradicate mosquito nests.	3 points for knowing to: return home after an order from the authorities, dispose of food contaminated with flood water.	2 points for knowing to return home after an order from the authorities.	1 point for knowing nothing

*Source:* Aksa, F.I., Ashar, M., Siswanto, H.W. & Malem, Z.Z., 2025, ‘Immersive virtual reality for improving flood evacuation behaviour and self-efficacy’, *Jàmbá: Journal of Disaster Risk Studies* 17(1), a1655. https://doi.org/10.4102/jamba.v17i1.1655

Meanwhile, the self-efficacy questionnaire was developed from previous research conducted by D’Amico et al. (2023). Self-efficacy is a person’s belief in their ability to complete difficult tasks (Chichekian & Shore 2016; Newnham et al. 2017). Self-efficacy strongly influences a person’s behaviour and performance outcomes (Chichekian & Shore 2016; Newnham et al. 2017). To measure students’ self-efficacy in dealing with flood emergencies, the questionnaire was given to students before and after the learning session using SDG: Flood Alert! The questionnaire used a 1–5-point Likert scale with six statements (see Table 2).

**TABLE 2:** *T*-test results of knowledge-level comparison.

Variable	Test value = 0					
	*t*	*df*	Sig. (two-tailed)	Mean difference	95% CI of the difference	
					Lower	Upper
Knowledge before the flood	15 794	44	0.000	1.311	1.14	1.48
Knowledge during the flood	23 330	44	0.000	1.956	1.79	2.12
Knowledge after the flood	17 412	44	0.000	1.400	1.24	1.56

Sig., significance; *df*, degrees of freedom; CI, confidence interval.

In addition, this research also assessed the students’ intrinsic motivation after learning using SDG: Flood Alert! The questionnaire to measure intrinsic motivation was developed from previous research conducted by Lovreglio et al. (2022). Intrinsic motivation consists of five questions given after the learning session using the SDG: Flood Alert (Post-test).

### Data analysis

The data analysis in this research was carried out by normality test, homogeneity test and independent sample *t*-test using SPSS Version 27.0 statistical software.

### Ethical considerations

Ethical clearance to conduct this study was obtained from the National Research and Innovation Agency (BRIN) Republic of Indonesia and Lembaga Pengelola Dana Pendidikan (LPDP) Indonesia (Reference No. 495/UN54.6/PT.00.01/2025).

## Results

The results showed that *Serious Disaster Game: Flood Alert* effectively increases student knowledge (*T*-test, *p* < 0.005). There was an increase in knowledge score after learning using SDG *Flood Alert!* From the three aspects of knowledge assessed, scores significantly increased in the component of what to do during a flood (Pre-test *M* = 1.96 on a scale of 4 and Post-test *M* = 3.91 on a scale of 4). Most students were able to answer well on the actions that should be taken during a flood disaster (Table 3). This is because SDG *Flood Alert* product is designed using Kolb’s experiential learning cycle theory, which consists of several stages: Concrete experience, reflective observation, abstract conceptualisation (building working models and hypotheses) and active experimentation (new learning tried out in practice). For example, at level 1 (before the flood), players can take actions to reduce the impact of the flood, such as identifying evacuation routes, storing important documents and preparing a disaster preparedness kit. In the next level, players can also practice evacuation by not crossing undercurrents, waterways and puddles. The findings of this research corroborate with previous research which shows that game-based learning effectively improves knowledge and capacity in disaster risk reduction (e.g. D’Amico et al. 2023; Schueller et al. 2020; Tsai et al. 2019).

**TABLE 3:** Means and standard deviation of each aspect of knowledge.

Knowledge aspects	Pre-test		Post-test		
	*M*	s.d.	*M*	s.d.	*p*-value
What did you do before the flood?	1.87	0.450	3.27	0.440	< 0.005
What did you do during the flood?	1.96	0.420	3.91	0.280	< 0.005
What did you do after the flood?	1.80	0.450	3.11	0.420	< 0.005
Total	5.62	0.650	10.29	0.626	< 0.005

*Source:* Aksa, F.I., Ashar, M., Siswanto, H.W. & Malem, Z.Z., 2025, ‘Immersive virtual reality for improving flood evacuation behaviour and self-efficacy’, *Jàmbá: Journal of Disaster Risk Studies* 17(1), a1655. https://doi.org/10.4102/jamba.v17i1.1655

*M*, mean; s.d., standard deviation.

The advantage of the developed SDG Flood Alert product is that it provides a fun learning experience for students. They not only learn based on theory and conceptual knowledge, but are also involved in a flood disaster simulation. During the game, students must search for answers and determine what actions to take during a flood. This provides long-term retention for them when facing future disasters. Game-based learning strongly influences child development and enhances practical experience (Tsai et al. 2015). Turning knowledge into action is important in disaster risk reduction (Aksa et al. 2020a, 2020b; Aksa 2022). The SDG: Flood Alert is therefore a disaster learning media that combines knowledge and action by engaging directly in the game.

In addition, the use of SDG: Flood Alert also significantly affected students’ self-efficacy (*T, p* < 0.05).

There was an increase in scores after learning using SDG: Flood Alert (Table 3). Most students were confident that they could effectively deal with a flooding emergency (pre-test *M* = 2.31 and post-test *M* = 4.31). Students are also confident they will be able to deal with a flooding emergency even though the water level is critical, and the water speed does not allow it to move easily (Table 4).

**TABLE 4:** Self-efficacy analysis results.

Number	Questions	Pre-test self-efficacy		Post-test self-efficacy	
		Means	s.d.	Means	s.d.
1	I am confident that I can effectively deal with a flooding emergency	2.31	0.468	4.31	0.633
2	Thanks to my resources, I know how to manage in a flooding emergency	2.64	0.484	4.40	0.495
3	I would be able to deal with a flood emergency even if the water level were critical and the speed of the water did not allow me to move easily	2.49	0.506	4.09	0.51s
4	I would be able to cope with a flood emergency even if I found other people along the way	2.76	0.435	4.33	0.477
5	I would be able to cope with a flood emergency even if the exit were blocked and the water level did not allow me to open the doors and go out.	2.80	0.405	4.31	0.468
6	I would be able to deal with flood emergency even if I found objects that could injure me along the way	2.82	0.387	4.11	0.418

*Source:* Aksa, F.I., Ashar, M., Siswanto, H.W. & Malem, Z.Z., 2025, ‘Immersive virtual reality for improving flood evacuation behaviour and self-efficacy’, *Jàmbá: Journal of Disaster Risk Studies* 17(1), a1655. https://doi.org/10.4102/jamba.v17i1.1655

s.d., standard deviation.

The post-test results also show that students know how to handle flood emergencies (*M* = 4.40 on a scale of 5) (Table 4). This increase in self-efficacy is because SDG: Flood Alert is designed to present various challenges during a flood. Players are directed to evacuate by not passing through undercurrents or puddles and not touching electrically charged objects. The game scenario effectively increases students’ confidence and readiness to face floods.

The findings of this research corroborate previous researches, which found that using serious games as interactive learning media significantly increases self-efficacy and disaster preparedness (Chittaro & Sioni 2015; Feng, Duives & Hoogendoorn 2021a; Feng et al. 2021b). Students who have high self-efficacy tend to take protective actions in the face of disasters (Chittaro & Buttussi 2015). Those with high self-efficacy believe they can take the right action or decision when a disaster occurs. This is because self-efficacy is an important indicator of individual decision-making during emergencies (Liu et al. 2023; Newnham et al. 2017; Yip et al. 2013).

The SDG: Flood Alert presents an engaging learning scenario for students to face danger that can depict actual flood conditions. This can train them to take appropriate and effective actions to manage better flood disaster events. The results show that students are motivated and confident to face flood emergencies.

Meanwhile, related to intrinsic motivation, most students enjoy learning using SDG: Flood Alert (*M* = 4.40 on a scale of 5). Most students also mentioned that SDG: Flood Alert is very interesting (*M* = 4.06 on a scale of 5).

Only a small number mentioned that SDG: Flood Alert is boring (*M* = 1.80 on a scale of 5) (Table 5). The high intrinsic motivation among students after using SDG: Flood Alert is because the SDG gameplay is designed interactively; a player can walk in four directions and can interact, such as picking up or storing objects. Gameplay SDG: Flood Alert allows players to explore the surrounding environment and complete mission commands.

**TABLE 5:** Intrinsic motivation.

Number	Statements	Mean	s.d.
1	I enjoy learning with the Serious Disaster Game (SDG): Flood Alert!	4.40	0.495
2	Serious Disaster Games (SDG): Flood Alert! are fun to perform	4.33	0.477
3	Serious Disaster Games (SGs): Flood Alert! are boring	1.80	0.45
4	Serious Disaster Games (SDG): Flood Alert! does not hold my attention at all	2.31	0.468
5	Serious Disaster Games (SDG): Flood Alert! as very interesting	4.06	0.53

s.d., standard deviation.

Based on these findings, SDG: Flood Alert can be used as an interactive learning media for students. The SDG: Flood Alert can be an alternative solution to overcome the limitations of disaster learning using a conventional module-based approach.

## Conclusion

In the context of disasters, experience has a significant effect on preparedness. However, not all people have had direct disaster experiences. This study concluded that SDG: Flood Alert plays a crucial role in increasing students’ behaviour during flood evacuations. It can be further concluded that SDG: Flood Alert is effective in increasing students’ knowledge, self-efficacy and intrinsic motivation. The SDG: Flood Alert provides an enjoyable learning experience for students, which prepares and empowers them to respond effectively during flood events. Students not only learn theory and conceptual knowledge, but are also involved in disaster simulations, which train students’ decision-making skills. SDG: Flood Alert learning can provide long-term retention for students. This study further concluded that its findings corroborate with previous researches, which found that game-based learning is effective in increasing students’ capacity for disaster risk reduction. This research highlights that turning knowledge into action is an important component of disaster risk reduction.
